# The pleiotropic functions of GOLDEN2-LIKE transcription factors in plants

**DOI:** 10.3389/fpls.2024.1445875

**Published:** 2024-08-19

**Authors:** Mengyi Zheng, Xinyu Wang, Jie Luo, Bojun Ma, Dayong Li, Xifeng Chen

**Affiliations:** ^1^ College of Life Sciences, Zhejiang Normal University, Jinhua, China; ^2^ National Engineering Research Center for Vegetables, Beijing Vegetable Research Center, Beijing Academy of Agriculture and Forestry Science, Beijing, China

**Keywords:** GOLDEN2-LIKEs (GLKs), transcription factor, function, signalling pathway, in plants

## Abstract

The regulation of gene expression is crucial for biological plant growth and development, with transcription factors (TFs) serving as key switches in this regulatory mechanism. GOLDEN2-LIKE (GLK) TFs are a class of functionally partially redundant nuclear TFs belonging to the GARP superfamily of MYB TFs that play a key role in regulating genes related to photosynthesis and chloroplast biogenesis. Here, we summarized the current knowledge of the pleiotropic roles of GLKs in plants. In addition to their primary functions of controlling chloroplast biogenesis and function maintenance, GLKs have been proven to regulate the photomorphogenesis of seedlings, metabolite synthesis, flowering time, leaf senescence, and response to biotic and abiotic stress, ultimately contributing to crop yield. This review will provide a comprehensive understanding of the biological functions of GLKs and serve as a reference for future theoretical and applied studies of GLKs.

## Introduction

**Figure 1:**
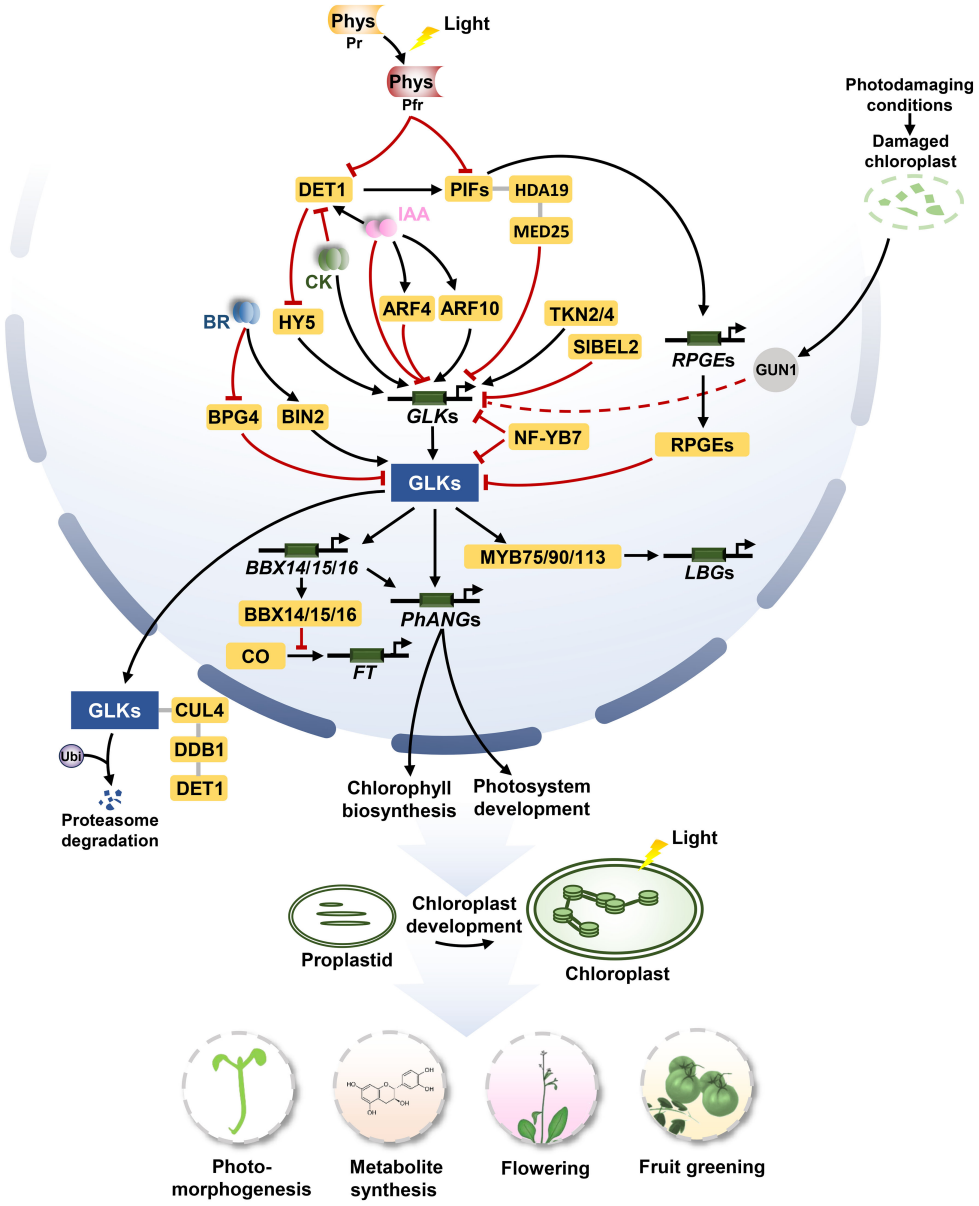
The signaling pathways of *GLK*s in regulating chloroplast biosynthesis, photomorphogenesis, flowering, and metabolite synthesis. For chloroplast biogenesis, *GLK*s activate the expression of *PhANGs* to promote the development of chloroplast. TKN2 and TKN4 activate the expression of *GLK2*, while BEL2 negatively regulates the expression of *GLK2* to promote the establishment of the ‘green shoulder’ in tomato fruits. ARF10 directly induces the expression of *GLK1* and ARF4 inhibits the transcription of *GLK1*. For photomorphogenesis, activated phytochromes (Phys) repress PIF and DET1 under light conditions. DET1 promotes the stability of PIF1 proteins, meanwhile, it mediates the proteasome degradation of GLK by interacting with CUL4 and DDB1 to form a ubiquitin ligase complex. The PIF1/PIF3-HDA19-MED25 complex reduces transcriptional repression of *GLK1* under light conditions. Activated BIN2 phosphorylates and thus stabilizes GLKs under light conditions. BPG4 suppress the transcription activity of GLKs via inhibition to their DNA-binding ability. HY5 binds the promoter of GLKs, inducing their activities to promote chloroplast development. Under dark conditions, PIFs can directly bind to the GLK1 promoter to repress the expression of *GLK1*. Moreover, PIFs activate the expression of *RPGE*s. RPGEs interact with GLKs to disrupt the DNA-binding activity of GLKs. In photodamaging conditions, the activity of GUN1 appears to down-regulate the expression of *GLK1* when plastids are dysfunctional. For flowering, GLKs directly activate the expression of *BBX14*, *BBX15* and *BBX16*, and the BBX proteins physically interact with the circadian clock regulator protein CO in the nucleus, which prevents CO-mediated *FT* transcription from repressing flowering. For metabolite synthesis, GLK1 interacts with the MBW complexes MYB75/90/113 and activates the transcriptional activity to enhance the expression of genes related to anthocyanin-specific biosynthetics including *LBG*s. Arrows and lines with end lines indicate positive regulation and negative regulation, respectively. Grey lines indicate interaction. Dashed arrow represents indirect effects through unknown intermediate factors.

**Figure 2:**
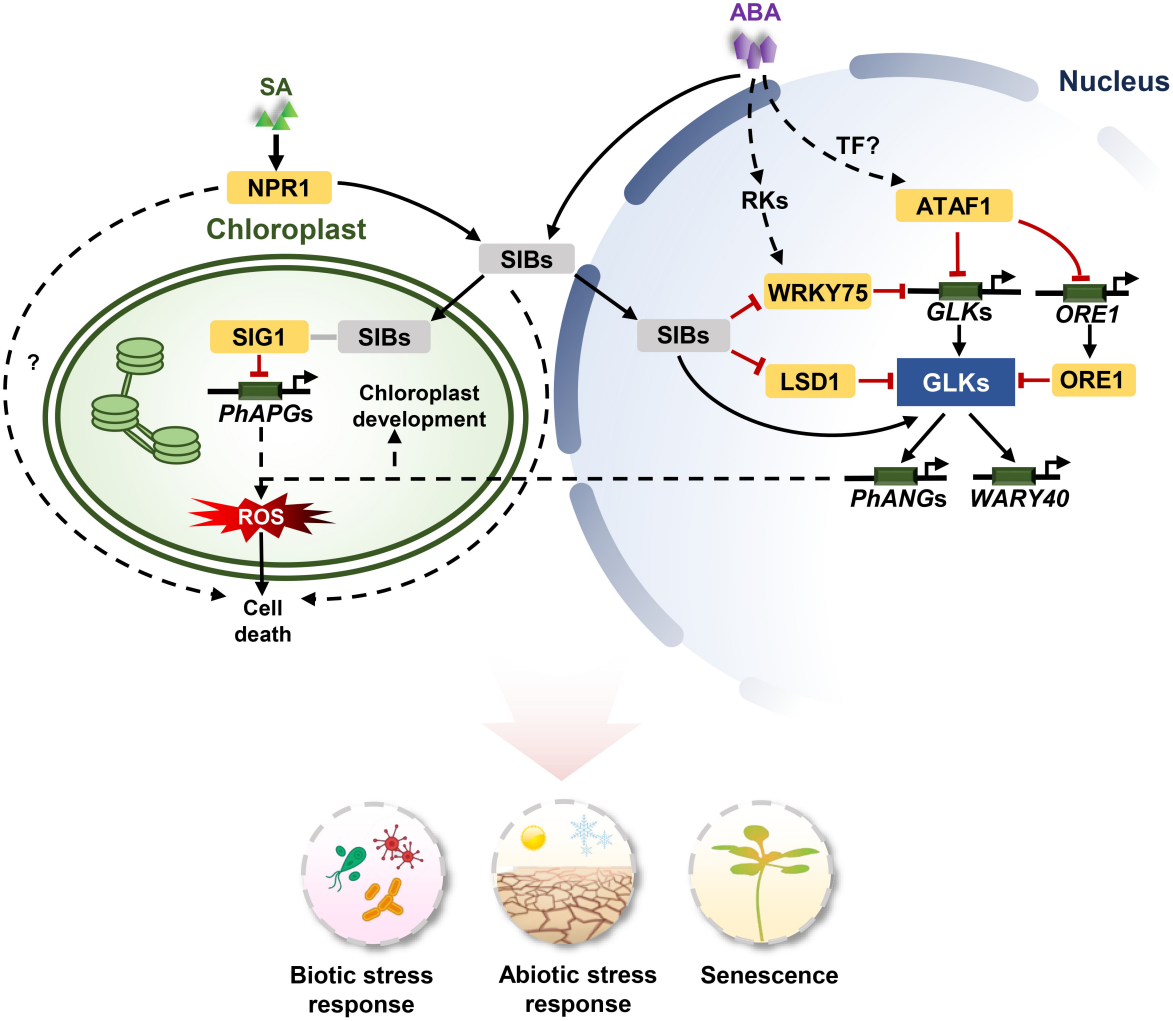
The signaling pathways of *GLK*s in stress response and senescence. For biotic stresses, SA-mediated NPR1 activation leads to the expression of *SIB1*. SIB1 proteins are targeted to both the nucleus and chloroplasts. SIB1 interacts with SIG1 to inhibit *PhAPG*s expression in chloroplasts, and SIB1 activate *GLK*s to induce the expression of *PhANG*s in the nucleus. The uncoupled expression of *PhANG*s and *PhAPG*s leads to an increase of ^1^O_2_ and PQH_2_ levels in chloroplasts. The reactive oxygen species (ROS) burst contributes to cell death. LSD1 antagonistically regulate the expression of *GLKs* with *SIB1* and functions in cell death. For abiotic stress, For abiotic stress, SIBs are induced by ABA and interact with WRKY75 to inhibit its transcriptional function. WRKY75 directly binds to the promoters of *GLKs* to repress their expression. ATAF1 responds to ABA and suppresses the expression of *GLK1* by directly binding to the promoters of *GLK1* and *ORE1*. ORE1 interacts with GLK1 to inhibit its transcriptional activity. *ATAF1* expression is regulated by unknown upstream TFs. ABA activates *GLK*s via core ABA signalling components PYL/PYRs-PP2Cs-SnRKs, and subsequently GLKs induce the expression of *WRKY40*. Arrows and lines with an end line indicate positive regulation and negative regulation, respectively. Grey lines indicate interaction. Dashed arrows represent indirect effects through unknown intermediate factors.

GOLDEN2-LIKEs (GLKs) are plant-specific transcription factors (TFs) involved in multiple biological processes in plants (Chen et al., 2016; Lambret-Frotte et al., 2023). GLKs are members of the GARP superfamily, containing a nuclear localization signal, a DNA-binding domain (DBD), a proline-rich domain and a GLK/C-terminal (GCT) box (Riechmann et al., 2000; Safi et al., 2017). The DBD consists of three α-helices followed by a highly conserved motif of AREAEAA, which confers specific characteristics to GLKs and distinguishes GLKs from other GAPR members (Fitter et al., 2002). To date, GLKs are widespread in land plants, and the last common ancestor of GLKs might be from Embryophyta (Wang et al., 2013; Hernández-Verdeja and Lundgren, 2023). GLKs are demonstrated to be the key regulators for chloroplast biogenesis from lower plants to higher plants (**Table 1**; **Figure 1**). Additionally, mounting evidence shows that the GLKs also function in multiple aspects through the entire lifetime of plants, including seedling photomorphogenesis, hormone signalling, leaf senescence, flowering, fruit nutrition and bio- or abiotic stress responses (**Table 1**; **Figures 1**, **2**). GLKs might be a node of signaling networks in plants, which are valuable to research for crop improvement in molecular breeding.

**Table 1:** Informations and functions of *GLK*s in plants.

Function	Plant souce	Gene name	Defend against targets	Method	Overexpression host plants	Governance mode	Reference
Chloroplast development	*Zea mays* (Maize)	*ZmGLK1/2*	/	OE, KO	Rice	+	(Li et al., 2020b; Yeh et al., 2022)
	*Arabidopsis thaliana* (Arabidopsis)	*AtGLK1/2*		OE, KO	Arabidopsis, Tomato	+	(Fitter et al., 2002; Waters et al., 2009; Kobayashi et al., 2012; Powell et al., 2012)
	*Physcomitrium patens* (Moss)	*PpGLK1/2*		Homologous recombination	/	+	(Yasumura et al., 2005)
	*Oryza sativa* (Rice)	*OsGLK1/2*		OE, KO	Rice	+	(Nakamura et al., 2009; Wang et al., 2013)
	*Solanum lycopersicum* (Tomato)	*SlGLK1/2*		OE, KO	Tomato	+	(Nguyen et al., 2014; Niu et al., 2022)
	*Capsicum annuum* (Pepper)	*CaGLK2*		Co-localized with *pc10*	/	+	(Brand et al., 2014)
	*Brassica napus* (Rapeseed)	*BnaGLK1*		OE	*Brassica napus*	+	(Pan et al., 2017; Zhang et al., 2024a)
	*Arachis hypogaea* (Peanut)	*AhGLK1*		OE, RNAi	Peanut	+	(Liu et al., 2018, 2020)
	*Prunus persica* (Peach)	*PpGLK1*		OE, VIGS	Arabidopsis	+	(Chen et al., 2018)
	*Actinidia chinensis* (Kiwifruit)	*AchGLK*		OE	Tomato	+	(Li et al., 2018)
	*Malus domestica* (Apple)	*MpGLK1*		OE	Arabidopsis	+	(An et al., 2019; Yang et al., 2023)
	*Betula platyphylla × B. pendula* (Hybrid birch)	*BpGLK1*		OE, RNAi	Hybrid birch	+	(Gang et al., 2019)
	*Lactuca sativa* (Lettuce)	*LsGLK*		CACTA transposon occurred, Complementation test	/	+	(Zhang et al., 2022b)
	*Populus alba × P.berolinensis* (Hybrid poplar)	*PabGLKs*		OE, RNAi	Hybrid poplar		(Li et al., 2021)
	*Hordeum vulgare* (Barley)	*HvGLK1/2*		OE, KO	Barley	+	(Taketa et al., 2021)
	*Camellia sinensis* (Tea plant)	*CsGLK1/2*		OE	Tomato	+	(Wang et al., 2022)
	*Marchantia polymorpha* (Liverwort)	*MpGLK1*		OE, KO	Liverwort	+	(Yelina et al., 2024)
	*Raphanus sativus* (Radish)	*RsGLK2.1*		OE, KO	Arabidopsis	+	(Ying et al., 2023)
	*Catharanthus roseus* (Catharanthus roseus)	*CrGLK*		VIGS, Chloroplast retrograde signaling inducers	/	+	(Cole-Osborn et al., 2024)
	*Liriodendron chinense × L. tulipifera* (*Liriodendron* hybrids)	*LhGLK1*		OE	Arabidopsis	+	(Qu et al., 2024)
Fruit quality	*Solanum lycopersicum* (Tomato)	*SlGLK1/2*		OE	Tomato	+	(Nguyen et al., 2014)
	*Oryza sativa* (Rice)	*OsGLK1/2*		OE	Rice	+	(Li et al., 2022c)
	*Actinidia chinensis* (Kiwifruit)	*AchGLK*		OE	Tomato	+	(Li et al., 2018)
	*Arabidopsis thaliana* (Arabidopsis)	*AtGLK1/2*		OE	Tomato, Arabidopsis		(Powell et al., 2012; Sun et al., 2022)
	*Camellia sinensis* (Tea plant)	*CsGLK1/2*		OE	Tomato	+	(Wang et al., 2022)
Flowering	*Arabidopsis thaliana* (Arabidopsis)	*AtGLK1/2*		OE, KO	Arabidopsis	–	(Waters et al., 2009; Susila et al., 2023)
	*Liriodendron chinense* × *L. tulipifera* (*Liriodendron* hybrids)	*LhGLK1*		OE	Arabidopsis	–	(Qu et al., 2024)
Leaf senescence	*Arabidopsis thaliana* (Arabidopsis)	*AtGLK1/2*		OE, KO	Arabidopsis	–	(Rauf et al., 2013)
	*Brassica napus* (Rapeseed)	*BnaGLK1a*		OE, RNAi	Rapeseed	–	(Zhang et al., 2024a)
Biotic stress responses	*Arabidopsis thaliana* (Arabidopsis)	*AtGLK1/2*	*Fusarium graminearum*	OE	Arabidopsis	+	(Savitch et al., 2007)
			*Botrytis cinerea*	OE, KO		+	(Murmu et al., 2014)
			*Hyaloperonospora arabidopsidis Noco2*	OE, KO		+	(Savitch et al., 2007)
			*Pseudomonas syringae pv. tomato*	KO		–	(Wang et al., 2017a)
			*Cucumber mosaic virus*	KO		+	(Han et al., 2016)
	*Arachis hypogaea* (Peanut)	*AhGLK1b*	*Pseudomonas syringae pv. tomato*	OE	Peanut	+	(Ali et al., 2020)
	*Nicotiana benthamiana* (Tobacco)	*NbGLK1*	*Potato virus X*	OE	Tobacco	+	(Sukarta et al., 2020)
	*Oryza sativa* (Rice)	*OsGLK1*	*Rice black-streaked dwarf virus*	OE, KO	Rice	+	(Li et al., 2022a)
Abiotic stress responses	*Arabidopsis thaliana* (Arabidopsis)	*AtGLK1/2*	Ozone	OE	Arabidopsis	+	(Nagatoshi et al., 2016)
			High light	OE, KO		+	(Zeng et al., 2023; Li et al., 2023b)
			Osmotic and dehydration	OE, KO		–	(Ahmad et al., 2019)
	*Arachis hypogaea* (Peanut)	*AhGLK1*	Drought	OE	Arabidopsis	+	(Liu et al., 2018)
	*Gossypium hirsutum* (Cotton)	*GhGLK1*	Cold, drought	OE	Arabidopsis	+	(Liu et al., 2021)
	*Zea mays* (Maize)	*ZmGLK1/2*	Drought	OE	Rice	+	(Li et al., 2023a)
			High light				(Li et al., 2020b)

OE, Overexpression; RNAi, RNA interference; VIGS, Virus-induced gene silencing; KO, Gene knockout; “+”, Positive regulation; “-”, Negative regulation.

## GLKs control chloroplast biogenesis and function maintenance

Chloroplast is an important place for photosynthesis in plants (Jarvis and López-Juez, 2014). Solid evidence indicated that GLKs control chloroplast biogenesis by transcriptionally targeting photosynthesis-related nuclear genes (*PhANG*s), including chlorophyll biosynthesis and photosynthesis-related genes (Waters et al., 2009; Martín et al., 2016). Constitutive expression of *GLK*s could increase chloroplast numbers and chlorophyll content in photosynthetic tissues, such as leaves or fruits (Nguyen et al., 2014), and even in non-photosynthetic tissues such as roots and callus in Arabidopsis (*Arabidopsis thaliana*) (Nakamura et al., 2009; Kobayashi et al., 2012). In tomato (*Solanum lycopersicum*), the expression of *GLK2* gradiently reduced from the shoulder to the base in fruit, which influences a gradient of chloroplast development of fruit forming the ‘green shoulder’ fruits (Powell et al., 2012; Nguyen et al., 2014). The TFs KNOTTED1-like Homeobox (KNOX) TKN2 and TKN4 activate the expression of *GLK2* to promote the establishment of ‘green shoulder’ fruit in tomato (Nadakuduti et al., 2014). However, BEL1-like HOMEODOMAIN 2 (BEL2) affects the formation of ‘green shoulder’ in tomato fruits by negatively regulating the gradient expression of *GLK2* (Niu et al., 2022). In addition, *GLK*s were affected by AUXIN RESPONSE FACTORs (ARFs) in regulating chlorophyll accumulation in tomato fruit (Sagar et al., 2013; Yuan et al., 2018). In rice (*Oryza sativa*), a member of the nuclear factor Y (NF-Y) TF family, OsNF-YB7, inactivates the transactivation activity of GLK1 at multiple regulatory layers to inhibit chlorophyll accumulation in the embryo of rice (Yang et al., 2024). In radish (*Raphanus sativus*), GLK2 interacts with NUCLEAR FACTOR Y, SUBUNIT A 9a (NF-YA9a) to increase the expression of the chlorophyll biosynthesis gene, *RsHEMA2*, which improves the chloroplast development (**Figure 1**; Ying et al., 2023).

Interestingly, *GLK*s are functionally redundant in C_3_ plants. In Arabidopsis and rice, the *glk1* or *glk2* single mutant has no phenotypic difference from the wild type (WT), and the *glk1*/*glk2* double mutant displayed pale green leaves and abnormal chloroplast structure (Fitter et al., 2002; Wang et al., 2013). However, the functional redundancy of *GLK*s does not exist in the C_4_ plant. For instance, maize (*Zea mays*) *glk2* single mutant showed yellow leaves with abnormal chloroplast structure (Rossini et al., 2001). It is well known that the chloroplasts become different between the C_3_ and the C_4_ plants, the former has only one type of chloroplast in mesophyll cells (MC), while the latter has two types of chloroplasts in the bundle sheath cells (BSC) and the MC, respectively (Majeran et al., 2009). The development of chloroplasts in the BSC provides an anatomical basis for efficient photosynthesis in C_4_ plants (Miyake, 2016). In C_4_ plants such as maize and sorghum (*Sorghum bicolor*), *GLK1* expressed much more in MC than that in BSC, while *GLK2* expressed more in BSC contrarily (Wang et al., 2013; John et al., 2014). In addition, the tissue-expression pattern of *GLK1* and *GLK2* are almost similar in Arabidopsis (**Supplementaryray Figure S1**), but different in maize (**Supplementary Figure S2**). It was considered that both GLK orthologs retained the ability to induce chloroplast biogenesis and play important roles in regulating the differentiation of chloroplast development in C_4_ plants (Rossini et al., 2001), but recent studies showed that *GLK2* adopted a more prominent developmental role, particularly in relation to chloroplast activation in BSC (Lambret-Frotte et al., 2023).

To maintain the functional stability of chloroplasts in plants, the chloroplast-to-nucleus retrograde signalling (RS) is essential for coordinating the expression of *PhANG*s and photosynthesis-associated plastid genes (*PhAPG*s; Pogson et al., 2008). Defective chloroplasts in mutants of plastid protein emphasize coordination between chloroplastic protein processing and nuclear transcription (Chan et al., 2016). GENOMES UNCOUPLED1 (GUN1), a chloroplast-localized pentatricopeptide-repeat protein, is a central integrator participating in multiple RS pathways. In photodamaging conditions, the activity of GUN1 appears to down-regulate the expression of *GLK1* when plastids are dysfunctional (Kakizaki et al., 2010); GUN1/GLK1 module represses the expression of *B-box structural domain PROTEIN16* (*BBX16*) to regulate the well-established expression of *PhANG*s (**Figure 1**; Veciana et al., 2022). However, aside from the GUN1/GLK1 module, studies also showed that the ubiquitin-proteasome system participates in the degradation of Arabidopsis GLK1 in response to plastid signals in a GUN1-independent manner (Tokumaru et al., 2017).

## GLKs modulate the photomorphogenesis of seedlings

Seedling photomorphogenesis is coordinately processed as inhibition of hypocotyl elongation, the opening of cotyledon, and chloroplast development when exposed to light. In Arabidopsis, *GLK*s are induced by light (Fitter et al., 2002). The *glk1*/*glk2* double mutant displayed decreased chlorophyll content, longer hypocotyls and less separated cotyledons (Martín et al., 2016; Alem et al., 2022). PHYTOCHROME-INTERACTING FACTORs (PIFs) are central regulators of photomorphogenesis in plants (Leivar and Monte, 2014). PIFs can form a complex with the histone deacetylase HDA19 and the Mediator subunit MED25, thus attenuating the transcriptional repression of *GLK1* by binding to the PBE motif (CACATG) on *GLK1* promoter in darkness (Martín et al., 2016; Guo et al., 2023), while light-activated phytochrome reverses this activity, thereby inducing *GLK*s expression (Martín et al., 2016). Interestingly, PIFs can also induce the expression of the *REPRESSOR OF PHOTOSYNTHETIC GENES 1* (*RPGE1*) and *RPGE2* in darkness, and then the RPGEs inhibit the DNA-binding activity of GLK1 by disrupting its dimerization, revealing another mechanism of PIF-mediated GLK repression (Kim et al., 2023). Besides, rice Phytochrome-Interacting Factor-Like1 (OsPIL1), a basic helix-loop-helix transcription factor, is also involved in the promotion of chlorophyll biosynthesis (Sakuraba et al., 2017). Moreover, DEETIOLATED 1 (DET1), a repressor of light-induced photomorphogenesis, not only promotes the protein stability of PIF1 (Shi et al., 2015), but also interacts with GLKs and promotes the degradation of GLK proteins by ubiquitination (Tang et al., 2016; Zhang et al., 2024b). Another regulator of photomorphogenesis, ELONGATED HYPOCOTYL5 (HY5) not only directly activates the expression of *GLK*s, but also interacts with the GLK proteins, suggesting that HY5 might first activates the expression of *GLK*s promote chlorophyll biosynthesis and photosystem formation, and then interacts with GLK proteins to inhibit hypocotyl elongation (Zhang et al., 2024b). Furthermore, indole-3-acetic acid (IAA) and cytokinin (CK) regulate *GLK2* in the opposing directions at the transcriptional level in a HY5-dependent manner to regulate chlorophyll biosynthesis in Arabidopsis roots (Kobayashi et al., 2012).

Additionally, the transcription factor, TEOSINTE BRANCHED 1, CYCLOIDEA, and PROLIFERATING CELL FACTOR 15 (TCP15), participates in the expression of *PhANG*s and binds to the same promoter regions of target genes as GLK1. It is postulated that GLK1 helps to recruit TCP15 for coordinating the expression of cell expansion genes with that of genes involved in the development of the photosynthetic apparatus (Alem et al., 2022). A regulator involved in BR signalling, BRASSINOSTEROID INSENSITIVE2 (BIN2), regulates physically interacts with and phosphorylates GLKs, and this phosphorylation stabilizes and activates GLKs to promote chloroplast development and photomorphogenesis (Zhang et al., 2021). Conversely, BRZINSENSITIVE-PALE GREEN 4 (BPG4) inhibits the transcriptional activity of GLKs by interacting with the GCT-box of GLKs and plays an inhibitory role in regulating chloroplast development and homeostasis (**Figure 1**; Tachibana et al., 2024).

## GLKs participate in the synthesis of metabolites

Photosynthetic products of chloroplasts generally contribute to the accumulation of carbohydrates, lycopene, carotenoids or other nutrient related substances in fruits (Klee and Giovannoni, 2011; Jia et al., 2020). Interestingly, GLKs can interact with the G-box Binding Factor (GBF) and activate the transcription of *PHYTOENE SYNTHASE* (*PSY*), promoting the biosynthesis of carotenoids (Sun et al., 2022). Overexpression of the exogenous *GLK*s increases the contents of carbohydrates, carotenoids, and tocopherol (vitamin E) in fruits of tomato (Powell et al., 2012; Nguyen et al., 2014; Lupi et al., 2019). Endosperm-specific overexpression of rice *GLK1* promotes the biosynthesis of carotenoids in the endosperm (Li et al., 2022c). Ectopic overexpression of the *GLK* homolog from pepper (*Capsicum annuum*), kiwifruit (*Actinidia chinensis*), and tea (*Camellia sinensis*) in tomato resulted in higher levels of carotenoids and sugar in the ripened fruits (Brand et al., 2014; Li et al., 2018; Wang et al., 2022). In addition, *GLK*s induce the biosynthesis of secondary metabolites including catechin and anthocyanin. *CsGLK*s are also involved in light-regulated catechin accumulation in tea plants by regulating the expression of *CsMYB5b* (Wang et al., 2022). In Arabidopsis, GLK1 interacts with the WD40-BHLH-MYB (MBW) complexes MYB75/90/113 and activates the transcriptional activity to enhance the expression of genes related to anthocyanin-specific biosynthetic including *late biosynthesis genes* (*LBG*s) (Li et al., 2023b). Meanwhile, *GLK2* activates the expression of *LBG*s and *TRANSPARENT TESTA GLABRA 1* (*TTG1*) through AtHY5-mediated light signalling and positively regulates anthocyanin biosynthesis in Arabidopsis (**Figure 1**; Liu et al., 2022; Zeng et al., 2023).

## GLKs negatively regulate flowering time and leaf senescence

The flowering time of plants is tightly controlled by endogenous or exogenous signals (Bouché, et al., 2016). It was reported that chloroplasts RS regulated flowering mediated by the floral repressor *FLOWERING LOCUS C* (*FLC*) in Arabidopsis (Feng et al., 2016). GLK1 and GLK2 act as downstream components of the chloroplast RS pathway that negatively regulates flowering time. The *glk1*/*glk2* double mutant of Arabidopsis displays early flowering, and overexpression of *AtGLK1*, *AtGLK2* or *LhGLK1* in Arabidopsis delayed flowering time (Waters et al., 2009; Qu et al., 2024). GLKs directly activate the expression of *BBX14*, *BBX15* and *BBX16*, and these BBXs proteins physically interact with the circadian clock regulatory CONSTANS (CO) in the nucleus, which prevent CO-mediated *FLOWERING LOCUST* (*FT*) transcription and repress flowering (**Figure 1**; Susila et al., 2023).

The chloroplast displays early signs of senescence symptoms, including a decrease in chlorophyll and a decline in photosynthetic efficiency (Soudry et al., 2005). *PIF3*, *4*, and *5* are up-regulated during age-triggered and dark-induced leaf senescence, and the accumulation of PIFs protein inhibits the expression of *GLK*s to impair chloroplast development and chlorophyll biosynthesis, leading to leaf senescence (Song et al., 2014). In addition, *GLK*s also respond to abscisic acid (ABA) in regulating plant senescence. The ABA pathway generally promotes leaf senescence, while GLKs negatively modulate ABA-mediated leaf senescence. Both *SIB*s and *WRKY75* are upregulated during leaf senescence and induced by ABA. SIBs interact with WRKY75 and thereby repress its transcriptional function, thus negatively regulating ABA-induced leaf senescence in a WRKY75-dependent manner. In contrast, WRKY75 positively modulates ABA-mediated leaf senescence in a GLK-dependent manner by directly binding to the W-box (T/CTGACC/T) in the *GLK*s promotor and inhibits their expressions (Zhang et al., 2022a; Lee et al., 2023). In addition, ABA can activate a NAC transcription factor ATAF1, which activates *ORESARA1* (*ORE1*) and represses *GLK1* expression by directly binding to the promoters of both genes. ORE1 also interacts with GLKs to inhibit the transcriptional activity of GLK1, resulting in impairing the expression of GLK target genes and leaf senescence (**Figure 2**; Rauf et al., 2013; Garapati et al., 2015). In *Brassica napus*, *GLK1a* has also been shown to directly influence the ABA signalling pathway. Overexpressing *BnGLK1a* delayed the leaf senescence upon ABA treatment (Zhang et al., 2024a).

## GLKs are involved in biotic and abiotic stress response

Current studies have shown that GLKs participate in the defence response of plants. The *glk1*/*glk2* double mutant of Arabidopsis showed enhanced resistance to *Pseudomonas syringae* pv. *tomato* and *Hyaloperonospora arabidopsidis* (Wang et al., 2017a). However, overexpression of *AtGLK1* contributes to inducing the expression of *pathogenesis*-*related* (*PR*) genes, which in turn confers resistance to *Fusarium graminearum* (Savitch et al., 2007). Additionally, overexpression of *AtGLK1* enhances the resistance to *Botrytis cinerea* in a jasmonic acid (JA)-independent manner, while increasing the susceptibility to *Hyaloperonospora arabidopsidis* Noco2 in a JA-dependant manner (Savitch et al., 2007; Murmu et al., 2014). *GLK*s play positive roles in resistance to *cucumber mosaic virus* (*CMV*), the *Potato virus X* (*PVX*), the *rice black-streaked dwarf virus* (*RBSDV*) and the *maize rough dwarf disease* (*MRDD*) (Han et al., 2016; Sukarta et al., 2020; Li et al., 2022b; Xu et al., 2023). Nevertheless, the virulence protein P69 of *Turnip yellow mosaic virus* (*TYMV*) interacts with GLKs and suppresses GLKs transcriptional activity, affecting the normal growth of plants and causing disease symptoms (Ni et al., 2017). Salicylic acid (SA) is an important hormone that regulates the defence responses to environmental stresses and against pathogens in plants (Kunkel and Brooks, 2002). LESION-SIMULATING DISEASE 1 (LSD1) is an SA-induced cell death regulator and a negative regulator that inhibits the DNA-binding activity of GLK1 towards its target promoters, and SIB1 proteins appeared to interrupt the LSD1-GLK interaction, and the subsequent SIB1-GLK interaction activated EX1-mediated singlet oxygen (^1^O_2_) signalling, leading to cell death and stress response in plants (Li et al., 2022a).

In addition, GLKs actively participate in the response to abiotic stresses. *AhGLK1* upregulates the expression of *AhPORA* during recovery from drought in peanuts (*Arachis hypogaea*), stimulating chlorophyll biosynthesis and photosynthesis to increase the survival rate from drought (Liu et al., 2018). Virus-induced silencing of *GhGLK1* in cotton (*Gossypium hirsutum*) leads to a great impact on growth and yield under drought and cold stress, and *GhGLK1* helps to increase the adaptability of Arabidopsis in drought and cold stress (Liu et al., 2021). Overexpression of maize *GLK* genes in rice improves light harvesting efficiency via Photosystem II (PSII), thus buffering the adverse effects of photoinhibition under high or fluctuating light conditions (Li et al., 2023a). In addition, GLKs affect ABA sensitivity and ion channel activity of plants to regulate stomatal movements under stresses. The ABA-responsive genes *WRKY40* is regulated by GLKs to increase the sensitivity of seedlings to osmotic stress, and the core ABA signalling components, PYL/PYRs-PP2Cs-SnRKs, possibly act as the intermediary in GLKs-induced *WRKY40* expression (Ahmad et al., 2019). In Arabidopsis, the chimeric repressors for GLKs (GLKs-SRDX) downregulate the genes for inwardly rectifying K^+in^ channels and K^+in^ channel activity to close the stomata to enhance the tolerance to ozone (Nagatoshi et al., 2016). Recently, the role of GLKs in various abiotic stress responses has been predicted in multiple species through genome-wide analysis, including soybean (*Glycine max*), millet (*Setaria italica*), bamboo (*Phyllostachys edulis*), orange (*Citrus sinensis*) and western balsam poplar (*Populus trichocarpa*) (Alam et al., 2022; Chen et al., 2022; Wu et al., 2022; Xiong et al., 2022; Wu et al., 2023). These facts indicate a broad and conserved function in the abiotic stress response of *GLK*s in plants, which awaits further validation.

## Molecular breeding application of *GLK*s in crops

Improving plant photosynthesis efficiency is an effective strategy for high-yield breeding in crops. Mounting evidence indicates that manipulation of *GLK*s achieves yield improvement in plants. In Arabidopsis, leaf-specific and silique wall-specific promoters were used to drive high expression of *AtGLK1*, resulting in enhanced leaf and silique wall photosynthesis and increased seed oil content by 2.88% and 10.75%, respectively (Zhu et al., 2018). In *B. napus*, overexpression of *BnGLK1a* resulted in a 10% increase in the thousand-seed weight of rapeseed (Zhang et al., 2024a). These results suggest that *GLK*s are promising tools for improving seed yield and oil production in oilseed crops.

Since the photosynthesis efficiency of C_4_ plants is much higher than that of C_3_ plants (von Caemmerer et al., 2012), the ectopic expression of maize (C_4_ plant) *ZmGLK*s was carried in rice (C_3_ plant) to improve its yield. The engineering rice plants induced chloroplast development in BSC accompanied by the accumulation of photosynthetic enzymes and intercellular connections (Wang et al., 2017b; Yeh et al., 2022). Overexpression of the *ZmGLK1* and *ZmGLK2* in rice increased the yield by 30% to 40% (Li et al., 2020b), while expression of *ZmGLK*s driven by its native promoter in rice increased the yield by 47% to 70% (Yeh et al., 2022).

## Discussion

GLK is a key regulator of chloroplast development. Knockout of *GLK*s lead to abnormal chloroplast structure but not complete distortion of chloroplast biogenesis (Fitter et al., 2002; Wang et al., 2013), suggesting the existence of other genes which can partly compensate for GLKs function in chloroplast development. Besides, though GLKs are considered to play important roles in regulating the differentiation of chloroplast development in C_4_ plants (Rossini et al., 2001), the molecular mechanism remains unclear. Recently, it was shown that the pleiotropic role of GLKs beyond chloroplast regulation, including photomorphogenesis, synthesis of secondary metabolites, flowering, senescence and response to biotic and abiotic stresses (**Table 1**). Regarding *GLK*s being functionally redundant in chloroplast development in C_3_ plants, it’s natural to think whether *GLK*s are also redundant in regulating other aspects of life. Clarifying these questions would be helpful in understanding the bio-function of *GLK* in plants.

As core regulators in plant, GLKs are involved in multiple molecular modes of action including response to upstream genes, binding to downstream target genes and protein-protein interactions. However, so far, some studies only proved the interaction relationship between GLK and target proteins. The specific binding elements still await further research. The expression of *GLK* can be regulated by the upstream regulators by binding to specific *cis*-elements in the promoter, such as T/CTGACC/T (W-box), CACGTG (G-box) or CACATG (E-box) (Zhang et al., 2022a; Sakuraba et al., 2017). Besides, GLK can also bind to the promotor of target genes downstream to regulate their expression. The highly conserved motif CCAATC is considered a widely shared *cis*-acting element for downstream targets of GLKs (Waters et al., 2009). Comparative cross-species analyses of GLKs have shown that most of the binding sites of GLKs were species-specific (Tu et al., 2022), providing support for further exploration of binding sites rich in downstream targets of GLKs in the future. Furthermore, the DNA-binding domain and GCT-box of GLK proteins are specific binding domains for most regulatory factors. Interestingly, a few proteins also bind to proline-rich regions of GLK proteins, such as LSD1 (Li et al., 2022a). As for the degradation, SlGLK2 is proven a substrate of the CULLIN4 (CUL4) - UV-DAMAGED DNA BINDING PROTEIN 1 (DDB1) - DET1 ubiquitin ligase complex for the proteasome degradation (Tang et al., 2016). However, the ubiquitin-proteasome system is also shown to participate in the degradation of Arabidopsis GLK1 in response to plastid signals (Tokumaru et al., 2017). Would it also be a part of the ‘CUL4-DDB1-DET1 degradation pathway’? Further research is needed to clarify their relationship.

In addition, GLKs have shown a rosy application prospect. By regulating the gene expression of *GLK*s, not only can the photosynthetic efficiency of crops be increased which in turn improves crop yields, but leaf morphogenesis can also be changed. It makes GLKs potentially applicable to agronomic trait improvement, horticultural plant breeding and ornamental plant improvement. However, overexpression of *GLK*s has certain negative effects. For example, transgenic rice of *ZmG1* drived by the constitutive promoter resulted in reduced seed size and no increase in yield (Yeh et al., 2022). Overexpression of *OsGLK1* in rice causes abnormal tapetum development and low seed setting rates, and also increased endosperm chalkiness of rice grains (Zheng et al., 2022; Li et al., 2022c). To mitigate the potential negative effects, the expression level of *GLK*s may be tightly regulated by selecting appropriate promoters, or ‘Knock-up’ by gene-editing techniques (Lu et al., 2021; Wang et al., 2024). Accurate regulation of the expression of GLKs will help improve crop overall quality and bring breakthroughs in agricultural production.
