# Supplementary material for: The pleiotropic functions of GOLDEN2-LIKE transcription factors in plants
Source: Front Plant Sci. 2024 Aug 19;15:1445875. doi: 10.3389/fpls.2024.1445875 (PMC11366661; doi:10.3389/fpls.2024.1445875)
Supplement: Supplementary file 1 [file Presentation1.pptx]

## Slide 1
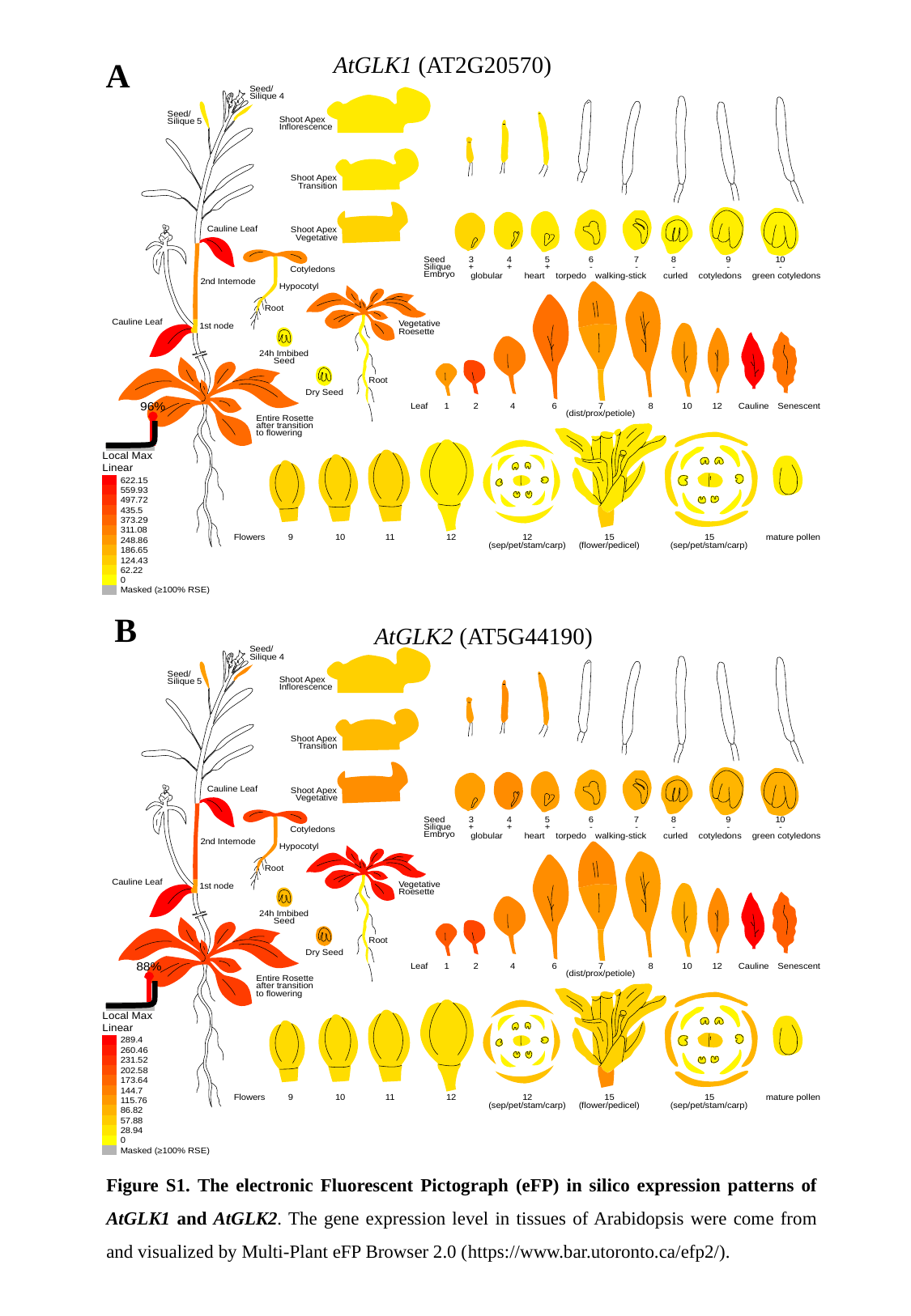

AtGLK1 (AT2G20570)
A
B
AtGLK2 (AT5G44190)
Figure S1. The electronic Fluorescent Pictograph (eFP) in silico expression patterns of AtGLK1 and AtGLK2. The gene expression level in tissues of Arabidopsis were come from and visualized by Multi-Plant eFP Browser 2.0 (https://www.bar.utoronto.ca/efp2/).

## Slide 2
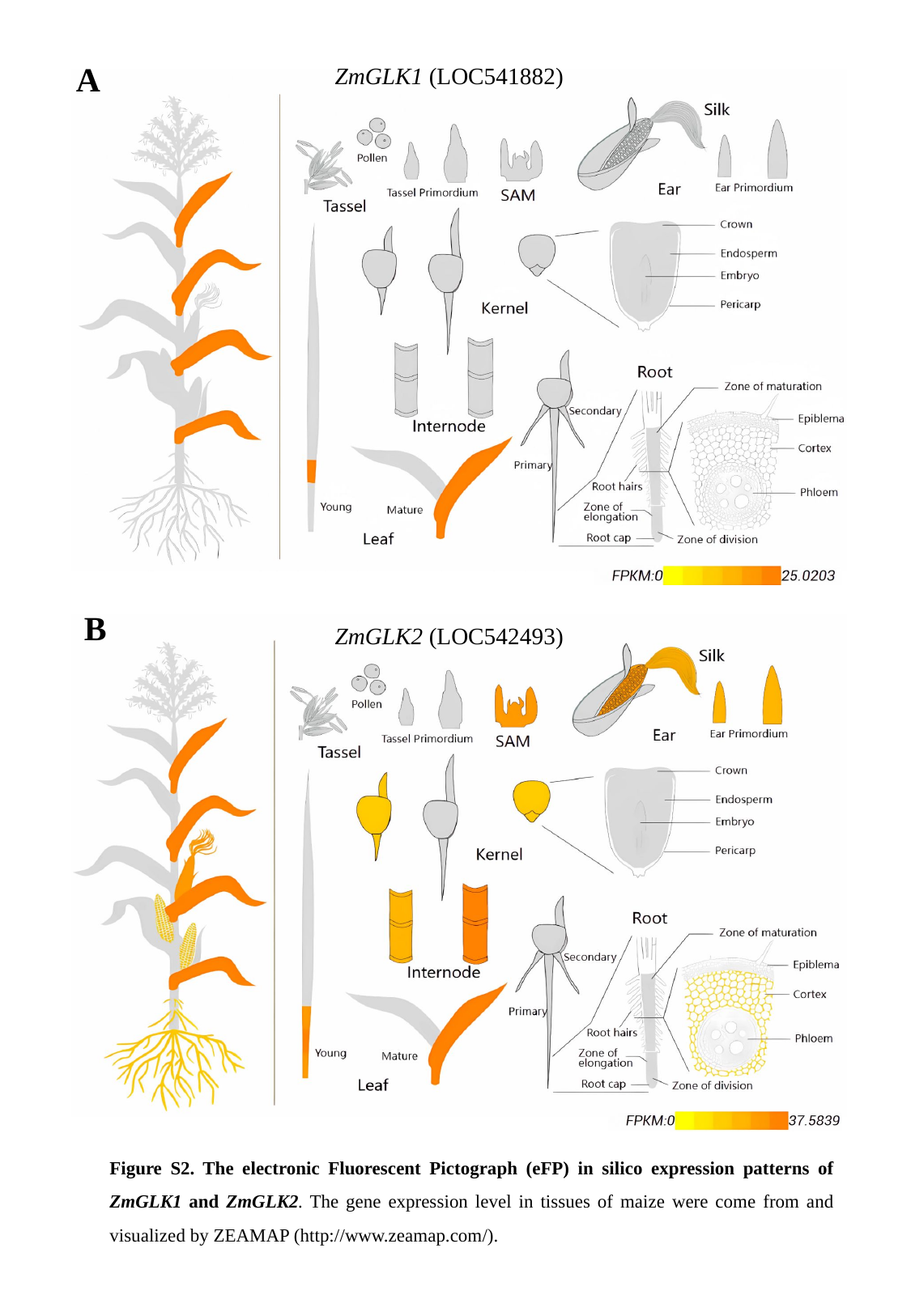

A
ZmGLK1 (LOC541882)
B
ZmGLK2 (LOC542493)
Figure S2. The electronic Fluorescent Pictograph (eFP) in silico expression patterns of ZmGLK1 and ZmGLK2. The gene expression level in tissues of maize were come from and visualized by ZEAMAP (http://www.zeamap.com/).
